# One third of hospital costs for atherothrombotic disease are attributable to readmissions: a linked data analysis

**DOI:** 10.1186/1472-6963-14-338

**Published:** 2014-08-08

**Authors:** Emily R Atkins, Elizabeth A Geelhoed, Matthew Knuiman, Tom G Briffa

**Affiliations:** School of Population Health, University of Western Australia, 35 Stirling Highway, Crawley, Western Australia Australia

**Keywords:** Cardiovascular disease, Coronary heart disease, Hospital, Peripheral vascular disease, Stroke, Linked data, Western Australia

## Abstract

**Background:**

Cardiovascular disease is the most frequent cause of death in Australia, with an associated cost burden of 11% of Australian annual health expenditure of which 40% is for hospital admissions. We investigated health outcomes and the components of hospital expenditure in the two years after an atherothrombotic disease admission to a tertiary hospital in an Australian setting.

**Methods:**

Using data linkage we analysed two years of hospitalisation data and death records of all men and women aged 35–84 years with an admission to a Western Australian tertiary hospital for atherothrombotic disease in 2007. Costs were identified by matching the Australian refined diagnostic related group on the admission records to the published schedules of public and private hospital costs for the period of interest, and converted to 2013 Australian dollars.

**Results:**

Of 6172 patients studied (74% coronary, 20% cerebrovascular, 6% peripheral), 783 (13%) died during follow-up and 174 of these were in hospital case-fatalities at index. Thirty-two percent of patients (n = 1965) accounted for 3172 readmissions to hospital with one in three having multiple hospitalisations. The hazard ratio of atherothrombotic disease readmission was 1.45 (95% CI 1.27, 1.66) in those with more than one vascular territory affected compared to those with only one territory affected after controlling for age, sex, comorbidity, admission type, procedures, and episode length of stay. The total index plus 2-year admission cost for atherothrombotic disease was calculated at $101 million; $71 million for index, and $30 million for readmissions.

**Conclusions:**

Among patients hospitalised with atherothrombotic disease, the cost of related rehospitalisations within 24 months is almost a third of the total. Much of the readmission costs fell within the first year. Whether readmissions and cost associated with atherothrombotic disease can be lowered through secondary prevention measures requires further investigation.

## Background

Cardiovascular disease (CVD) is the most frequent cause of death in Australia, and commands more of the government’s health expenditure than any other single disease. Approximately 11% of Australian annual health expenditure is spent on CVD, of which 40% is for hospital admissions [1]. Based on the latest health expenditure report (2010–2011 financial year) this equates to $14 and $5.7 billion respectively [2]. Better understanding of the hospitalisation costs associated with first and recurrent CVD events is central to evaluating the effectiveness of clinical care and planning for disease prevention.

The majority of CVD is attributable to atherothrombotic disease (ATD). ATD describes the shared pathology of atherosclerosis with superimposed thrombosis in ischaemic cerebrovascular disease (CeVD), coronary heart disease (CHD), and peripheral arterial disease (PAD); the combined determinants of CVD [3]. Atherothrombotic events in the cerebral and coronary arteries are life threatening and those occurring in the peripheral arteries are predominately limb threatening. Such events require urgent hospitalisation and intervention to prevent death and/or loss of a limb. Furthermore, secondary prevention is a vital part of ATD management as those who have experienced one atherothrombotic event are at high absolute risk of further manifestations of the disease [4–6]. But it is well understood that not all those who could benefit from secondary prevention pharmacotherapy receive it [7]. Work by Ademi et al. suggests that Western Australians are under-treated compared with the other Australian states [8]. Furthermore, analysis of pharmacy data by Gunnell et al. suggests that a large proportion of Western Australian patients with coronary heart disease are not filling scripts for all recommended therapeutic types in 28 days [9].

The two year medical costs of ATD have been modelled for eight European countries, [10] and 12- and 18-month costs have been modelled in another two [11, 12]. However, the two year hospital costs and outcomes of a cohort of patients hospitalised with ATD has yet to be described in an Australian context, particularly in terms of time from discharge. Our objective is to determine the health outcomes and estimate hospital costs for ATD in the two years after admission to a tertiary hospital in an Australian setting.

## Methods

### Study sample

The study sample comprised all Western Australian residents aged 35–84 years admitted to a tertiary public hospital in Perth during 2007 with a primary diagnosis of ATD selected using methods previously published [13]. In Western Australia (WA) the tertiary hospitals provide patients with acute and elective arterial interventions such as coronary angiograms and invasive vascular procedures, these were not available at outer metropolitan and rural public hospitals until quite recently, and as such tertiary hospitals were the only public hospitals that provided these services in the study period. At discharge, a letter is sent to the patient’s general practitioner (GP), if they have one, and a copy is given to the patient. The hospital doctor also has the option of seeing the patient in an outpatient clinic some time after discharge, especially if the patient does not have a GP. These services are not captured by the linked administrative data and are not included in this analysis.

The first ATD admission to a tertiary hospital in 2007 was identified and considered the index admission (this could be incident or recurrent ATD). Major comorbidities for ATD were also identified [13]. A history of ATD or comorbidity was recorded where so coded in any one of 21 diagnostic fields for any hospitalisation, including the index presentation, during the preceding 15 years [14]. Cardiovascular cause of death was defined by ICD 10 - AM code from I00 to I99.

### Data source

The data was extracted from two of the state’s administrative databases, the Hospital Morbidity Data Collection (HMDC) and Death Registrations. The HMDC covers all admissions to any public or private hospital in WA, while death registrations include those deaths registered in WA. Both databases are linked by the WA Data Linkage System at the Department of Health [15]. Data linkage is performed using probabilistic matching with clerical review [16]. The estimated proportion of invalid and missed links was 0.11% in 1996 [16] and linkage quality is expected to remain at this level for links between hospital and death records.

An episode of care refers to the period from a person’s first admission to the hospital system until they leave the hospital system. This may be an admission and discharge from the same hospital, or a contiguous set of transfers among hospitals. Rehospitalisation or readmission refers to episodes of care occurring after the index event. Rehospitalisation and death data were available for two years after the index event.

Ethics approval was obtained from the Human Research Ethics Committees at the WA Department of Health and The University of Western Australia.

### Costs

Costs were identified by matching the Australian refined diagnostic related group (AR-DRG) on the HMDC admission records to the published schedules of public and private hospital costs for the years from 2007 to 2009. We used AR-DRG version 5.1 public and private costs for admissions in 2007 and 2008, [17, 18] and the AR-DRG version 6.0 estimated public cost weights for admissions in 2009 [19]. No private costs have been published for 2009. Costs were then converted to constant 2013 Australian dollars using health index deflators [2].

Individual level costs for ATD episodes of care (defined as an ATD code in the primary diagnosis field at least once during the episode) were calculated on the index admission, and summed for readmissions up to 24 months following the index admission.

### Accessibility and remoteness and socio-economic disadvantage

The linked dataset included an Accessibility/Remoteness Index for Australia (ARIA+) and Socio-Economic Indices For Area (SEIFA) disadvantage score for each admission. ARIA + is based on distance by road to reach service centres [20]. Residence at the time of hospitalisation was categorised as: major city, inner regional, outer regional, remote, or very remote. This was further grouped as metropolitan (major city) and rural (inner regional, outer regional, remote, and very remote). SEIFA disadvantage scores are an area level measure based on census data relating to income, education, unemployment and motor vehicle ownership [21]. These scores are ranked and pooled into deciles, and were grouped into quintiles for this analysis. SEIFA disadvantage and ARIA + indices were missing for 270 (4%) patients.

### Statistical methods

Mean costs per individual for the index admission and for subsequent readmissions to 90 days, from 90 days to 12 months, and 12 to 24 months after index were calculated by 10-year age groups (35–44 years, 45–54 years, 55–64 years, 65–74 years, and 75–84 years at index admission), vascular territory, SEIFA disadvantage quintile, and ARIA + category of the index admission. Mean costs are presented as they reflect the population level costs. Chi-square test was used to compare the proportion of deaths in each age group.

Analysis of variance and t-tests were used to compare mean costs by age, sex, metropolitan or rural residence and socioeconomic disadvantage quintile. Cox regression was used to investigate factors associated with time to first ATD readmission within 2 years (and censored if died within 2 years). Age group, sex, rural or metropolitan residence, SEIFA disadvantage quintile, emergency or elective admission, vascular territory (incident or recurrent PAD, CeVD, or CHD), polyvascular disease (more than one territory affected), comorbidity history, and length of stay at the index admission were included in the base Cox model. Forward selection with significance level for entry set to 0.01 was used to check for interactions with age group, sex, polyvascular disease and index vascular territory. Statistical analysis was performed using SAS 9.4.

## Results

### Index admission

The sample comprised 6172 patients, the characteristics of which are presented in Table 1. Approximately two thirds were male, 79% were metropolitan residents and 21% in the most disadvantaged quintile. Coronary heart disease was the most common index diagnosis (n = 4571; 74%) and hypertension the most common comorbidity (n = 4009; 65%). Twenty-nine percent of patients had an ATD-related procedure at index, of these two-thirds had coronary stent procedures, 17% peripheral procedures, and 15% coronary artery bypass grafts.

**Table 1:** **Patient characteristics at index admission to tertiary hospital in 2007**

Characteristics	n = 6172
**Age, mean (SD)**	65.3952 (11.82)
**Age group, n (%)**	
35-44 years	321 (5.20)
45-54 years	912 (14.78)
55-64 years	1499 (24.29)
65-74 years	1758 (28.48)
75-84 years	1682 (27.25)
**Male, n (%)**	4036 (65.39)
**Episode length of stay, median (min, max)**	3 (1, 357)
**Accessibility and remoteness, n (%) n = 5902**	
Major city	4678 (79.26)
Inner regional	677 (11.47)
Outer regional	333 (5.64)
Remote	137 (2.32)
Very remote	77 (1.30)
**Australian socio-economic indices for area – Disadvantage, n (%) n = 5902**	
Most Q1	1243 (21.06)
Q2	1302 (22.06)
Q3	1336 (22.64)
Q4	1041 (17.64)
Least Q5	980 (16.60)
**Atherothrombotic disease subtype at index admission**	
Coronary heart disease, n (%)	4571 (74.06)
Cerebrovascular disease, n (%)	1242 (20.12)
Peripheral arterial disease, n (%)	359 (5.8)
**History of polyvascular disease at index admission**	768 (12.44)
**Comorbidities at index admission**	
Hypertension, n (%)	4009 (64.95)
Diabetes, n (%)	1683 (27.27)
Chronic kidney disease, n (%)	758 (12.28)
Atrial fibrillation, n (%)	1005 (16.28)
Heart failure, n (%)	862 (13.97)
Chronic lung disease, n (%)	764 (12.38)
Cancer, n (%)	1636 (26.51)

### Deaths

There were 783 (13%) deaths in the two years (including those occurring at the index event) and of these 439 had a cardiovascular cause of death code. Most deaths occurred in the first twelve months after the index event (n = 557; 71%). Of these, 174 (2.8% of total studied) were in-hospital case-fatalities at the time of the index event and another 85 out-of-hospital fatalities occurred in the 28 days following their index admission. The oldest age group experienced the greatest proportion of deaths with much smaller proportions in the younger age groups (35–44 years, n = 15, 1.92%; 45–54 years, n = 56, 7.15%; 55–64 years, n = 95, 12.13%; 65–74 years, n = 190, 24.27%; and 75–84 years, n = 427, 54.53%; p < 0.0001).

### Readmissions

Thirty-two percent (1965) of patients had at least one ATD readmission in the 2 years after the index admission and 1077 of those patients had a readmission in the first 90 days after the index admission*.* Fifty seven per cent of readmissions were emergency admissions. Table 2 shows the distribution of ATD readmissions and mean length of stay per admission by age group and overall.

**Figure 1:**
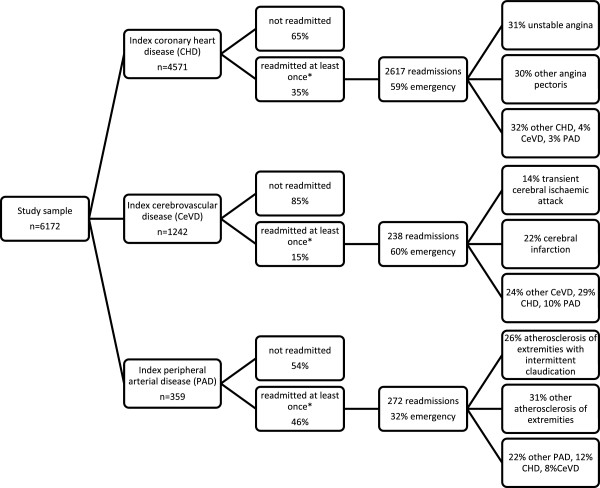
**Two year readmission characteristics of patients by vascular territory of index admission.** Branches may not add to 100% due to rounding *some patients have more than one readmission.

Figure 1 shows the pattern of readmissions over 2 years by type of index admission. Almost half of the patients with PAD were readmitted, mostly as elective cases. Whereas, a third of patients with CHD and one in seven with CeVD were readmitted and more so as emergency presentations. Readmissions involving another vascular territory were highest for stroke (39%), followed by PAD (20%) and CHD (7%). The proportions readmitted by index vascular territory was similar for each 10-year age group.

**Table 2:** **Number of recurrent ATD hospital admissions in two years from index admission, n (%), and mean length of stay per admission, days, within age group**

N^o^of recurrent admissions		0	1	2	3+	Total
**35-44 years**	**N (%)**	242 (75.4)	49 (15.3)	21 (6.5)	9 (2.8)	321
	**Mean length of stay**	-	7.2	4.2	4.1	5.3
**45-54 years**	**N (%)**	616 (67.5)	205 (22.5)	59 (6.5)	32 (3.5)	912
	**Mean length of stay**	-	7.7	3.6	2.8	5.3
**55-64 years**	**N (%)**	1021 (68.1)	311 (20.8)	100 (6.7)	67 (4.5)	1499
	**Mean length of stay**	-	4.0	3.8	4.8	4.2
**65-74 years**	**N (%)**	1168 (66.4)	399 (22.7)	117 (6.7)	74 (4.2)	1758
	**Mean length of stay**	-	4.7	5.8	4.0	4.8
**75-84 years**	**N (%)**	1160 (69.0)	355 (21.1)	96 (5.7)	71 (4.2)	1682
	**Mean length of stay**	-	8.8	7.4	4.6	7.0
**Total (%)**		**4207 (68.2)**	**1319 (21.4)**	**393 (6.4)**	**253 (4.1)**	**6172**

### Factors associated with time to ATD readmission

Having ATD in more than one vascular territory at the index admission was highly significant, with the risk of ATD readmission approximately 1.5 times higher in those with polyvascular disease compared to those with only one vascular territory affected controlling for age, sex, comorbidity, admission type, procedures, and episode length of stay (HR 1.45; 95% CI 1.27, 1.66). Rural or metropolitan residence and SEIFA disadvantage quintile were not included in the final model as they were non-significant and missing values were restricting the study sample included in the analysis. The risk of readmission was 2.3 to 2.6 times higher for incident or recurrent PAD than incident CHD for emergency index admissions, but not for elective index admissions (Table 3). The risk of readmission was 1.3 times higher for recurrent CHD than incident CHD for emergency, but not elective. The risk of readmission was lower for incident and recurrent CeVD than incident CHD, but not statistically significant for elective recurrent CeVD.

**Table 3:** **Cox hazard ratios for time to next ATD episode of care within 2 years by index admission vascular territory and index admission type (censored at death or 2 years)**

	Emergency	Elective	p-value
**Recurrent PAD**	2.61 (1.66, 4.10)	1.36 (0.99, 1.86)	0.0192
**Incident PAD**	2.29 (1.63, 3.22)	1.09 (0.81, 1.46)	0.0011
**Recurrent CeVD**	0.56 (0.40, 0.78)	0.62 (0.30, 1.26)	0.7973
**Incident CeVD**	0.42 (0.34, 0.51)	0.51 (0.29, 0.90)	0.4981
**Recurrent CHD**	1.29 (1.14, 1.46)	0.92 (0.77, 1.10)	0.0020
**Incident CHD**	1.00	1.00	-

In a Cox model with age group alone, the risk of readmission was lowest in the 35–44 years age group and all other age groups had similar risk (HR = 1.402 for 45–54 years, HR = 1.370 for 55–64 years, HR = 1.481 for 65–74 years, and HR = 1.460 for 75–84 years; all vs 35–44 years). However, in the fully adjusted model (polyvascular disease, index territory, sex, comorbidity, admission type, procedures, and episode length of stay), the risk of readmission tends to increase with age (HR = 1.290 for 45–54 years, HR = 1.265 for 55–64 years, HR = 1.348 for 65–74 years, and HR = 1.401 for 75–84 years; all vs 35–44 years).

### Costs

The total index plus 2-year admission cost for ATD was calculated at $101 million; $71 million for index (6172 episodes of care), and $30 million for readmissions, comprising $14 million in the 90 days after the index admission, $9 million from 90 days to 12 months, and $7 million from 12 to 24 months. Hospital admission costs were highly skewed with many patients not contributing readmission costs and a few patients contributing very high costs.

**Figure 2:**
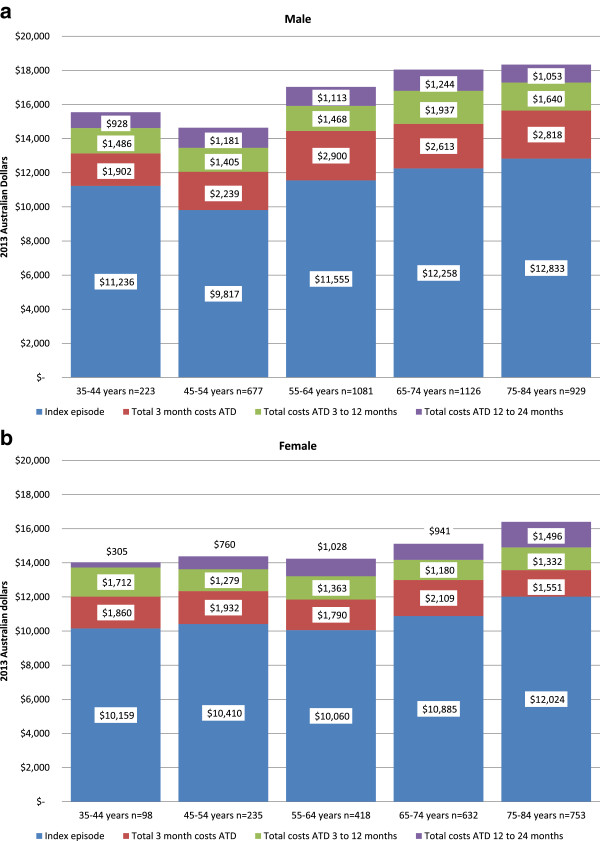
**Mean ATD admission costs per person at index (2007), 90 days, 1 year, and 2 years by sex (Panel a male and b female) and age group.** Costs are 2013 Australian dollars.

Figure 2 shows the mean ATD admission costs for men and women at each time point across five age groups. At index the mean cost of admission was not significantly higher for men ($11736, SD $14921) compared with women ($11039, SD $13846; p = 0.0673). However, readmission costs for ATD by sex at 90 days, 12 months were significantly higher for men, but not at 24 months follow-up (p < 0.0001; p = 0.0449; and p = 0.8586 respectively). There were no statistically significant differences in mean costs between age groups at each time point within males and females.

Index CeVD admissions had the highest mean cost ($18416, SD $19228), whilst patients with a PAD index admission had the highest mean readmission costs at 12 and 24 months (Table 4).

**Table 4:** **Mean and standard deviation of atherothrombotic disease admission costs per person at index admission and readmissions at 90 days, 90 days to 12 months and 12 to 24 months after index by vascular territory of index admission (2013 $AUD)**

	Index admission†	At 90 days after index admission‡	90 days to 12 months after index admission‡	12 to 24 months after index admission‡
**Coronary heart disease, mean (SD) ATD admission costs**	$9602 ($12249)	$2641 ($7866)	$1516 ($6291)	$1073 ($4836)
**Cerebrovascular disease, mean (SD) ATD admission costs**	$18416 ($19228)	$648 ($3702)	$875 ($4885)	$726 ($5396)
**Peripheral vascular disease, mean (SD) ATD admission costs**	$11652 ($15672)	$4576 ($11299)	$3746 ($10426)	$3172 ($15494)

†Cerebrovascular disease significantly different from coronary heart disease and peripheral arterial disease. ‡Significant differences between all vascular territories.

Emergency index admissions had the highest mean cost (Table 5). Of the readmission costs, the highest mean was for elective admissions within 90 days from index admission. Readmission costs decreased over time.

**Table 5:** **Mean and standard deviation of atherothrombotic disease admission costs by admission type per person using AR-DRGs adjusted to 2013 $AUD, rounded to whole dollars (n = 6172)**

	Index admission	At 90 days from index admission	90 days to 12 months from index admission	12 to 24 months from index admission
**Emergency admissions, mean (SD) ATD admission costs**	$12957 ($15152)	$858 ($4891)	$830 ($4642)	$703 ($4188)
**Elective admissions, mean (SD) ATD admission costs**	$8595 ($12422)	$1566 ($6041)	$725 ($4408)	$488 ($4701)

Rural residents, those in the inner and outer regional, remote and very remote ARIA + categories, had higher mean costs for the index tertiary hospital admission compared to major city residents ($13030 vs. $11088; p = 0.0001). Rural residents have a slightly longer, though not statistically significant, episode length of stay on the index admission. Readmission costs at each follow-up point were also slightly higher for rural residents, but the difference did not reach statistical significance.

The most disadvantaged socioeconomic quintile had the highest costs at index, 90 days, and 12 months (Table 6). At index and 24 months there were no statistically significant differences in mean costs between quintiles.

**Table 6:** **Mean and standard deviation of atherothrombotic disease admission costs by admission type per person using AR-DRGs adjusted to 2013 $AUD, rounded to whole dollars (n = 5902)**

Australian socio-economic indices for area – Disadvantage, mean (SD) ATD admission costs	Index admission	At 90 days from index admission†	90 days to 12 months from index admission†‡	12 to 24 months from index admission
**Most disadvantaged Q1**	$12118 ($15332)	$2855 ($9425)	$1934 ($7442)	$1077 ($3934)
**Q2**	$11543 ($14281)	$2282 ($7056)	$1587 ($6991)	$1140 ($5539)
**Q3**	$11035 ($13825)	$2339 ($6974)	$1578 ($6545)	$1279 ($8550)
**Q4**	$11417 ($14287)	$1991 ($6052)	$1254 ($5120)	$1303 ($6694)
**Least disadvantaged Q5**	$11322 ($15512)	$2192 ($7843)	$901 ($3888)	$827 ($4703)

Q = quintile †Significant differences between Q1 and Q5, and Q1 and Q4. ‡Significant differences between Q2 and Q5, and Q3 and Q5.

## Discussion

Of those admitted to a Perth tertiary hospital for ATD in 2007, 13% died and 32% experienced a readmission within 24 months. The 2-year cost to the health system, including index admission, was $101 million (2013 Australian dollars). Readmission costs for ATD in 24 months following an index admission were approximately 42% of the total, with more than three-quarters of these costs falling in the first 12 months after the index event.

A much greater proportion of those in the youngest age group had no readmissions, but there were remarkable similarities in the four older age groups and this cannot be explained by reduced follow-up in older age groups due to more deaths, as the estimated risks of readmission from the unadjusted Cox model showed a similar age pattern, reflecting the age-specific risks reported by Briffa et al. [22]. In the fully adjusted Cox model however there appeared to be an increasing risk of readmission with increasing age.

Those with PAD experienced a high proportion of readmissions. Almost half of the PAD group experienced at least one readmission, and 80% of their readmissions were for PAD. PAD-related hospital admissions are usually for advanced disease requiring costly vascular procedures. Those with PAD are known to be prescribed fewer cardio-protective medications than their counterparts who have experienced CeVD or CHD [8]. Improved use of secondary prevention drugs has the potential to reduce atherothrombotic change and delay or prevent hospital admissions for vascular procedures [23].

The international REACH registry reported that in three years of follow-up 28.4% of those with existing ATD experienced a vascular event or readmission after adjustment for age and sex, with those with CeVD experiencing the lowest readmission rates and PAD the highest [24]. We observed a similar pattern of lower readmissions in CeVD and higher in PAD, but our study population experienced a greater proportion of readmissions. This is likely due to the selection of our study sample based on admission proximity, whereas the international REACH registry recruited from general practice. The Oxford Vascular Study did have an acute ATD event as the baseline but the outcome of interest included acute events only, with 16 per cent having at least one recurrent acute event [25]. This population experienced a much higher proportion of CeVD (45%) and PAD (9%), and a lower proportion of CHD (42%). The large difference in CeVD may be attributed to the inclusion of those aged 85 years and older. The effects of polyvascular ATD and the index vascular territory on the risk of readmission for ATD are not unexpected. It likely reflects the use of vascular interventions and the complexity/severity of disease involving multiple territories.

The ATD hospital admission costs at 12 months for CHD, CeVD, and PAD are slightly higher than the mean hospital costs published by Ademi et al. in their analysis of the Australian REACH registry data in general practice patients [26]. Only 12% of their study sample experienced a hospital admission in the one year of follow-up and almost 11% of REACH participants had no prior ATD hospitalisation, suggesting a more clinically stable population.

A European analysis by Levy and colleagues also indicated that the vast majority of costs are incurred in the first year, though the costs varied substantially between the different countries [10]. The proportion of follow-up costs to acute costs for stroke in Europe ranged from 22% in Portugal to over 300% in France and Switzerland, though this included rehabilitation, which was not included in our study. For myocardial infarction it ranged between 33% in Portugal up to 114% in Austria.

The current study reports the costs associated with hospital readmissions for ATD in Perth Tertiary Hospitals. AR-DRGs have enabled measurement of the cost of ATD in such a large unselected sample. Indeed, a strength of the study is the bottom-up approach to costing by applying the individual AR-DRG costs to each admission in the dataset, based on the AR-DRG assigned in the HMDC. This means that the cost will best reflect the patient’s hospital admission and care costs. However, the costs assigned to AR-DRGs are based on an average patient across Australia and therefore may not reflect the precise costs incurred as a result of their admission.

## Conclusions

In conclusion, among patients hospitalised with ATD the total 2-year cost to the health system at $101 million is substantial. The costs of ATD-related rehospitalisation within 90 days of the index admission are also sizable, across age and sex categories, and readmission costs within 24 months is 42% of the baseline admission costs. With 3172 readmissions, and well-known prevention treatment gap, there is a need to further investigate the cost-effectiveness of secondary prevention pharmacotherapy as a potential means for reducing readmissions and costs, in this setting.

## Abbreviations

AM: Australian modification
AR-DRG: Australian refined diagnostic related group
ARIA+: Accessibility/Remoteness Index for Australia
ATD: Atherothrombotic disease
CeVD: Cerebrovascular disease
CHD: Coronary heart disease
CM: Clinical modification
CVD: Cardiovascular disease
HMDC: Hospital Morbidity Data Collection
ICD-10: The International Statistical Classification of Diseases and Related Health Problems, Tenth Revision
PAD: Peripheral arterial disease
SEIFA: Socio-economic Indices For Area
WA: Western Australia.
